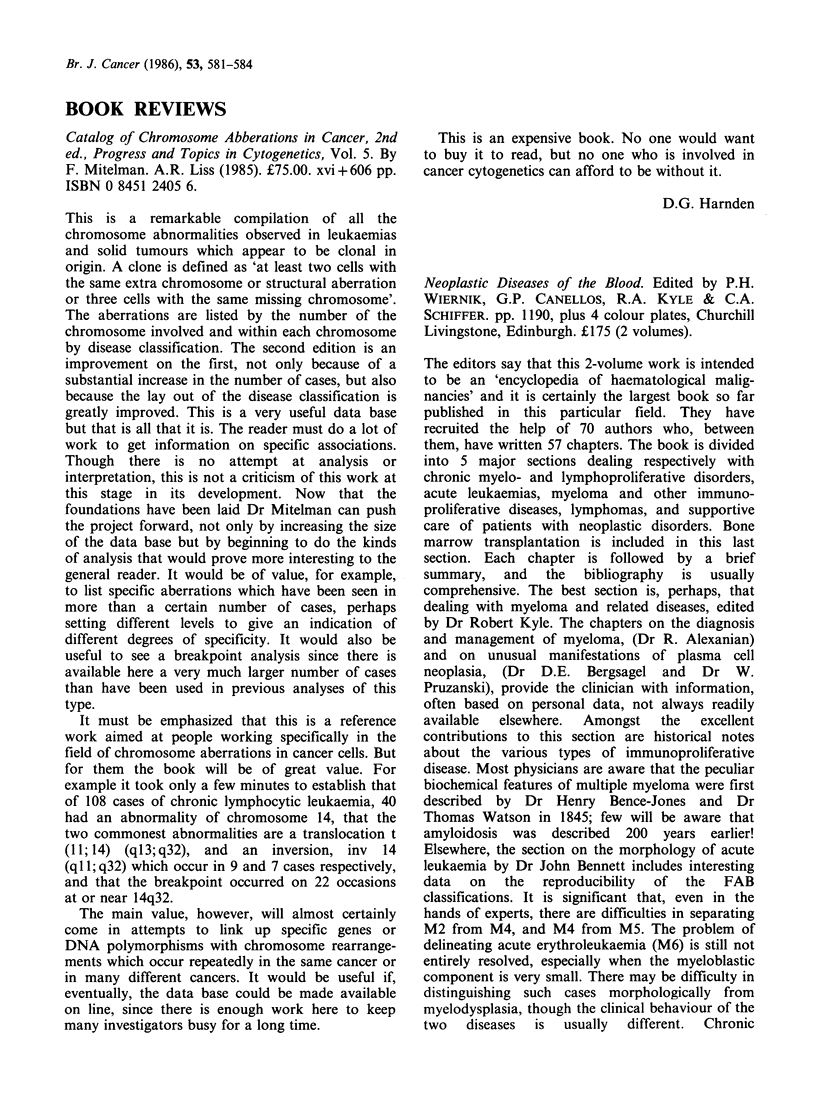# Catalog of Chromosome Abberations in Cancer, 2nd ed., Progress and Topics in Cytogenetics

**Published:** 1986-04

**Authors:** D.G. Harnden


*Br. J. Cancer* (1986), **53**, 581–584

### BOOK REVIEWS

*Catalog of Chromosome Abberations in Cancer, 2nd ed., Progress and Topics in Cytogenetics,* Vol. 5. By F. Mitelman. A.R. Liss (1985). £75.00. xvi + 606 pp. ISBN 0 8451 2405 6.

This is a remarkable compilation of all the chromosome abnormalities observed in leukaemias and solid tumours which appear to be clonal in origin. A clone is defined as 'at least two cells with the same extra chromosome or structural aberration or three cells with the same missing chromosome'. The aberrations are listed by the number of the chromosome involved and within each chromosome by disease classification. The second edition is an improvement on the first, not only because of a substantial increase in the number of cases, but also because the lay out of the disease classification is greatly improved. This is a very useful data base but that is all that it is. The reader must do a lot of work to get information on specific associations. Though there is no attempt at analysis or interpretation, this is not a criticism of this work at this stage in its development. Now that the foundations have been laid Dr Mitelman can push the project forward, not only by increasing the size of the data base but by beginning to do the kinds of analysis that would prove more interesting to the general reader. It would be of value, for example, to list specific aberrations which have been seen in more than a certain number of cases, perhaps setting different levels to give an indication of different degrees of specificity. It would also be useful to see a breakpoint analysis since there is available here a very much larger number of cases than have been used in previous analyses of this type.

It must be emphasized that this is a reference work aimed at people working specifically in the field of chromosome aberrations in cancer cells. But for them the book will be of great value. For example it took only a few minutes to establish that of 108 cases of chronic lymphocytic leukaemia, 40 had an abnormality of chromosome 14, that the two commonest abnormalities are a translocation t (11;14) (q13;q32), and an inversion, inv 14 (q11;q32) which occur in 9 and 7 cases respectively, and that the breakpoint occurred on 22 occasions at or near 14q32.

The main value, however, will almost certainly come in attempts to link up specific genes or DNA polymorphisms with chromosome rearrangements which occur repeatedly in the same cancer or in many different cancers. It would be useful if, eventually, the data base could be made available on line, since there is enough work here to keep many investigators busy for a long time.

This is an expensive book. No one would want to buy it to read, but no one who is involved in cancer cytogenetics can afford to be without it.

D.G. Harnden